# Tipping the balance: intricate roles of the complement system in disease and therapy

**DOI:** 10.1007/s00281-021-00892-7

**Published:** 2021-10-26

**Authors:** Richard B. Pouw, Daniel Ricklin

**Affiliations:** Molecular Pharmacy Group, Department of Pharmaceutical Sciences, University of Basel, 4056 Basel, Switzerland

**Keywords:** Complement, Inflammation, Autoimmune disease, Hemolysis, Complement therapeutics

## Abstract

The ability of the complement system to rapidly and broadly react to microbial intruders, apoptotic cells and other threats by inducing forceful elimination responses is indispensable for its role as host defense and surveillance system. However, the danger sensing versatility of complement may come at a steep price for patients suffering from various immune, inflammatory, age-related, or biomaterial-induced conditions. Misguided recognition of cell debris or transplants, excessive activation by microbial or damaged host cells, autoimmune events, and dysregulation of the complement response may all induce effector functions that damage rather than protect host tissue. Although complement has long been associated with disease, the prevalence, impact and complexity of complement’s involvement in pathological processes is only now becoming fully recognized. While complement rarely constitutes the sole driver of disease, it acts as initiator, contributor, and/or exacerbator in numerous disorders. Identifying the factors that tip complement’s balance from protective to damaging effects in a particular disease continues to prove challenging. Fortunately, however, molecular insight into complement functions, improved disease models, and growing clinical experience has led to a greatly improved understanding of complement’s pathological side. The identification of novel complement-mediated indications and the clinical availability of the first therapeutic complement inhibitors has also sparked a renewed interest in developing complement-targeted drugs, which meanwhile led to new approvals and promising candidates in late-stage evaluation. More than a century after its description, complement now has truly reached the clinic and the recent developments hold great promise for diagnosis and therapy alike.

## Complement: a fresh look at an old system

Portraying the complement system as a “novelty” may appear counterintuitive at best when considering both the ancient evolutionary origin of this innate immune branch, predating antibodies by millennia, and its initial description as host defense system that dates back to the dawn of the twentieth century [1, 2]. In a clinical setting, however, complement has only moved to the center of attention in the past decades, and the field of complement-targeted therapeutics is meanwhile evolving rapidly [3–5]. The shift in the perception of the complement system from auxiliary antimicrobial pathway to decisive pathological contributor and therapeutic target has been a long way coming and has been based on decades of seminal research that shed new light on molecular, functional, and clinical aspects of this fascinating protein cascade.

**Fig. 1:**
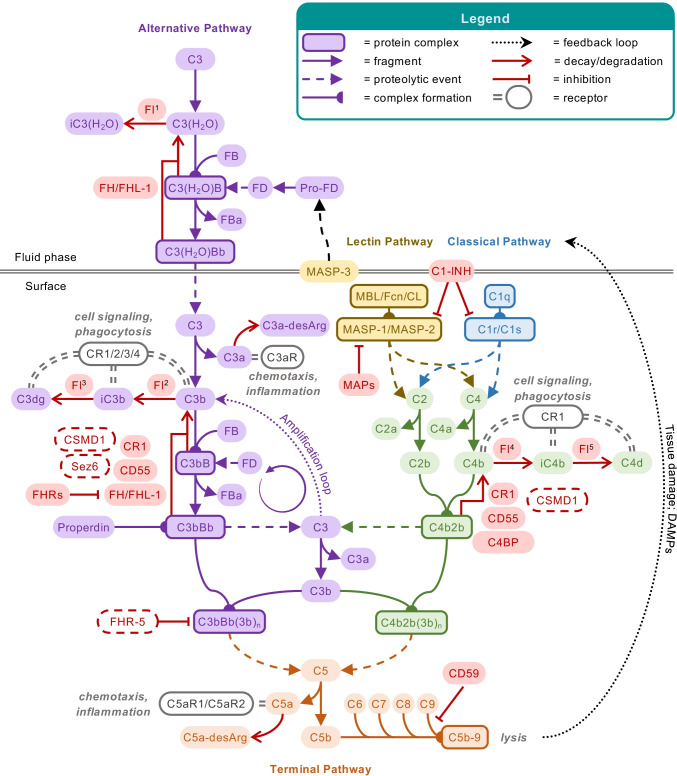
Schematic overview of the complement system. The complement cascade is initiated either via the fluid-phase formation of C3(H_2_O) (alternative pathway; AP) or through pattern recognition on a surface by either lectins (mannan-binding lectin; MBL, ficolins; Fcn, or collectins; CL) complexed with MASP-1 and MASP-2 (lectin pathway) or C1q complexed with C1r and C1s (classical pathway). All pathways lead to the formation of C3 convertases (C3bBb or C4b2b), which further cleave the central protein of the cascade, complement C3, into the small anaphylatoxin C3a and the larger fragment C3b. Deposition of the opsonins C4b and C3b on targeted surfaces mediates signaling through complement receptors (CR) on immune cells, induces cell activation, and facilitates phagocytosis. In parallel, C3b deposition perpetuates the cascade by forming new C3 convertases via the AP, also referred to as the amplification loop. This positive feedback loop leads to more C3b deposition that, at a sufficient density, turns C3 convertases into C5 convertases, which cleave C5 into the small anaphylatoxin C5a and the larger C5b. C5b is a nucleus for the formation of the membrane attack complex (MAC, C5b-9), via subsequent binding of C6, C7, C8, and multiple C9, forming the lytic pore. C5a and C3a induce chemotaxis and inflammatory responses via their C5a and C3a receptors (C5aR1/C5aR2 and C3aR, respectively) but are quickly degraded in plasma to their less potent forms C5a-desArg and C3a-desArg, respectively. The cascade is regulated at various steps, here indicated in red. FH and FHL-1 control the alternative pathway, whereas MAPs and C1-INH control the initiation of the lectin or classical pathway, with the latter acting on both. On the host surface, several additional regulators act on the formation of C3 convertases, as well as acting as co-factors for the degradation of the opsonins by FI. The extent of cleavage by FI depends on its co-factor: (1) C3(H_2_O) to iC3(H_2_O) requires FH/FHL-1; (2) C3b to iC3b occurs in the presence of FH, FHL-1, CD46 (or membrane cofactor protein; MCP), CR1 and the more recently described Sez6 protein family and CSMD1; (3) iC3b is further degraded to C3dg in the presence of CD46, CR1, Sez6, and CSMD1; (4) C4b is degraded to iC4b; and (5) subsequently C4d in the presence of C4BP, CR1, CD46, and CSMD1. While the remaining fragments can no longer perpetuate the complement cascade, they are still ligands for CRs. The decay of the convertases is accelerated by CD55 (or decay accelerating factor; DAF), CR1, CSMD1, FH/FHL-1, and Sez6 (C3b-based convertases only), and C4BP (C4b-based convertases only). The formation of the lytic ring by C9 is inhibited by CD59. New regulatory steps continue to be uncovered, including FHRs that might regulate FH/FHL-1, thereby preventing complement inhibition, whereas FHR-5 might add a new class of regulators to the system, acting specifically on C5 convertases. The extent of properdin stabilizing the C3b-based convertases remains topic of debate, possibly acting as a pattern recognition molecule for the alternative pathway or even comprising its own pathway in parallel to the three traditional ones. The role of MASP-3, a splice variant of the LP zymogen MASP-1, has only recently been appreciated, essentially enabling the maturation of pro-FD into FD

Perhaps the most essential change of dogma came with the realization that complement is not only employing its potent effector function for antimicrobial defense but may direct it to various endogenous and exogenous surfaces to confer broader immunosurveillance [6]. The sensing of molecular patterns, either pathogen- or damage-associated, often provides the trigger for inducing a cascade that marks threatening cells and facilitates their elimination via direct lysis, phagocytosis, and/or stimulation of downstream immune responses. Although the sensing of antibody clusters by C1q (i.e., classical pathway; CP) or microbial carbohydrate signatures by pattern recognition receptors of the lectin pathway (LP) are the best-known initiators, it becomes increasingly evident that the sensory capacity and spectrum of complement is much broader and includes altered-self signatures on apoptotic or hypoxic cells, among many others. Even without pattern recognition, the complement system targets surfaces via the spontaneous low-rate activation of its promiscuous alternative pathway (AP). Any surface attack by complement may lead to the formation of C3 convertases, which cleave the abundant plasma protein C3 into an anaphylatoxin (i.e., C3a) and an opsonin fragment (i.e., C3b) to induce effector functions (Fig. 1). When C3b is deposited on surfaces, it engages the constituents of the AP to form new convertases and drive a self-amplifying opsonization cycle. This inherent positive feedback loop increases C3b densities, and the C3 convertases redirect their activity toward the plasma protein C5, the cleavage of which generates another anaphylatoxin (i.e., C5a) and, via the C5b fragment, provides a nucleus for the formation of membrane attack complexes (MAC). While the lytic potential of MAC confers the most direct effector function and may lead to the killing of microbes, especially Gram-negative bacteria, few cells are in fact prone to direct lysis. In many cases, the broad receptor-mediated functions of complement opsonins, further supported by the inflammatory effect of the anaphylatoxins, drive the overall response. The release of C3a and C5a during activation leads to chemoattraction and priming of various immune cells (via signaling through anaphylatoxin receptors), whereas the interaction of opsonins with complement receptors (CR) on those cells induces shuttling to the lymphatic system (via CR1), stimulation of adaptive immune responses (via CR2), and phagocytosis (via integrin receptors CR3 and CR4). These receptor activities are also primarily responsible for complement’s impressive host defense crosstalk repertoire that ranges from platelet activation [7] and induction of coagulation responses [8] to the release of cytokines and modulation of T cell responses [9]. As the defensive actions of complement largely rely on its fast response time and broad activity, the sensing capacities can neither be highly specific nor are particularly fail-proof. Furthermore, the complement system does not contain a true negative feedback loop. This is in contrast to the other main protein cascade in circulation, *i.e.,* the coagulation system, where the activated thrombin-thrombomodulin complex cleaves protein C, which in turn leads to inhibition of pro-thrombin cleavage and limits overall coagulation [10]. The apparent lack of a direct negative feedback loop for complement might be expected when considering its focus on immediate microbial defense, where a self-limiting cascade might fall short of eliminating the treat. Without a negative feedback loop, the system solely relies on the presence of preformed complement regulators to keep its activation under control. Host cells therefore engage a panel of membrane-bound and soluble regulators that limit the action of initiating proteases and anaphylatoxins, interfere with opsonization, and amplification or prevent the formation of MAC (Fig. 1).

Traditionally, researchers and clinicians alike attempted to tightly link physiological and pathophysiological complement responses to individual pathways and/or effector functions. Meanwhile, however, a more refined and dynamic picture of complement functions emerges, in which the sum of all activating and regulating surface signatures and stimuli shape an overall response that typically involves several pathways and effectors and engages various crosstalk mechanisms [3]. Under physiological conditions, this tuned interplay of counteracting forces enables a differential response to various threat levels. For example, microbial infections require a more fulminant response that involves direct killing and phagocytic elimination alongside strong alert signaling and induction of downstream innate and adaptive immune responses. In contrast, the removal of apoptotic cells and debris necessitates a more delicate approach that includes limited opsonization and little to no inflammatory signaling to provide a ‘housekeeping’ function. Interestingly, it became evident that our bodies also employ this mechanism to confer homeostatic immunoediting during tissue development, most prominently by eliminating unwanted neuronal ends during synaptic pruning [11].

While the basic mechanisms of this well-oiled machinery have long been known, surprising new facets and players have only been identified, or fully appreciated, in recent years. On the initiation side, the lectin pathway gained a large share of attention due to the realization that the pattern recognition functions are not only exerted by mannose-binding lectin (MBL) but a full panel of ficolins and collectins with broad and distinct sensing profiles for carbohydrates but also acetylated structures and other signatures [12, 13]. At the same time, our comprehension of the interplay between the three MBL-associated serine proteases (MASPs) has improved, including a functional connection to yet unknown links with the alternative pathway [14–16], and new LP regulators have been described [17, 18]. Yet we also achieved a fine-grained understanding of mechanisms that drive classical pathway activation. Most importantly, structural insight revealed that density of antibody deposition and the consequent formation of antibody ‘platforms’ largely define the extent of immunoglobulin-mediated CP induction [19, 20]. Moreover, the isotype and glycan profile of the immunoglobulin and the protease loading on the pattern recognition protein C1q shape the response [21–24]. Yet, important new insight has also been gained on the regulatory side, particularly regarding the factor H (FH) family of complement regulators. Whereas FH itself is considered the main regulator of the AP, by recognizing self-surfaces, disassembling convertases, and enabling opsonin degradation, there is a growing appreciation of its splice variant, FH-like protein 1 (FHL-1) [25]. While the smaller size (seven instead of twenty domains) and reduced surface recognition capacity limits the regulators impact in circulation, the role of FHL-1 may be much more prominent in tissues due to its beneficial penetration profile [26]. An even larger change in perception could be observed for FH-related proteins (FHRs), which share surface- and opsonin-binding properties with FH but seem to lack its typical complement-regulatory capacities and may exist in dimeric form. As a consequence, FHRs may compete with FH for the same surfaces and possibly provide a ‘deregulation’ or fine-tuning step for the overall complement response [27]. The relation to a key regulator of complement activation sparked great interest in FHRs and rendered them the subject of extensive research over the recent years. Since their discovery, the clinical relevance of the FHRs has been well established, with numerous reports of aberrant FHRs associating with ‘typical’ complement-related diseases, including meningococcal disease [28], aHUS and other nephropathies [29–31], and AMD [32], among others. It is still unclear, though, whether the reported disease-associated mechanisms of FHRs reflect their normal function within the complement system. Apart from the likely role as FH competitors [33, 34], potentially with differential tissue specificity among the FHRs, distinct new functions of FHRs within the complement system and beyond may yet to be uncovered [35]. Furthermore, the deregulation of FH by FHRs may only be limited to surfaces, while not affecting the critical regulatory function of FH and FHL-1 on fluid-phase convertases. Notwithstanding such open questions, the presence of FHRs reveals a bigger picture that exceeds the classical dogma of sharply defined activator-regulator settings yet include surface- and context-dependent modulators such as FHRs but also pentraxins that may recruit activators and/or displace regulators to tailor the complement response. This notion of surface- and context-dependent regulation seems to become even more complex with new tissue-specific complement regulators entering the field [36, 37].

Such tuned reactivities become even more important when considering the second major dogmatic shift, as we now know that the hunting grounds of complement reach beyond the vasculature and include most tissues and potentially even intracellular spaces. The traditional view that complement components are solely produced by the liver, released into circulation, and act on blood-exposed cells has been replaced by far more holistic version. It is now well established that most nucleated cells are able to produce and secrete a broad set of complement components, some of which are even produced only extrahepatically [38, 39]. Local complement production may indeed be the driving force behind many physiological and pathological processes and become particularly important in secluded or immune-privileged tissues/organs such as the central nervous system [40, 41]. The availability of complement activators, regulators, and effectors may therefore be more ubiquitous than originally thought yet also highly distinct and dynamic, depending on the location and the environment. Even the perception of complement as strictly extracellular effector system has begun to totter in recent years. While the intracellular presence of complement components may not be surprising by itself when considering their previously mentioned extrahepatic production, the increasing association of cell-modulatory functions of intracellular complement, aptly termed ‘complosome’ [42], has stirred profound attention. Roles in cell homeostasis, differentiation and activation have meanwhile been assigned to several components, and their impact on physiological and pathophysiological processes is currently explored [43–45]. Whether the intracellular space allows canonical functions of complement or whether the role of the same component in the intra- and extracellular environment is unrelated entirely is matter of ongoing debate.

Finally, the ‘job description’ of complement is constantly extended as novel crosstalk mechanisms are reported at an impressive pace. Owing to the evolutionary age of complement and the resulting co-development with many host defense and homeostatic pathways, a connecting or even coordinating role could have been expected but has certainly not been fully appreciated until recently. The number and diversity of reported crosstalk functions have grown too large to be remotely covered in the scope of this summary, and we refer to focused review articles on this topic [46, 47]. What is important to remember conceptionally is that complement is among the earliest sensors of potential threats and directly acts on the surface of non-self (e.g., microbial intruders) or altered-self structures (e.g., apoptotic cells). Alongside lateral communication with other first-line-of-defense pathways such as the coagulation system, most of its crosstalk activity is directed downstream by inducing or propagating immune, inflammatory or homeostatic processes.

The fresh look at an ancient system therefore paints complement as an innate immune pathway that is far from acting as an isolated, monofunctional, locally restricted cascade system but rather as a highly dynamic, functionally broad, and ubiquitous immunosurveillance system that is employed in defense and tissue homeostasis. Under normal circumstances, complement exerts its important physiological functions without being noticed, and some of its roles are shared with or even taken over by other pathways, as in the case of the growing importance of adaptive immunity from childhood to adolescence. Conversely, any dysfunction or erroneous engagement of this potent effector system may easily result in an attack of host cells and contribute to various clinical complications.

## The thin line between defense and distress: complement’s pathological side

Although first reports of complement activation in disease can be traced back as far as the first decade of the 1900’s [48], and an involvement of complement in conditions such as rheumatoid arthritis or transplant rejection has long been considered [49], complement’s role as disease contributor has only reached the awareness of the broader clinical and pharmaceutical community with the therapeutic success of the anti-C5 antibody eculizumab (Soliris, Alexion) for the treatment of paroxysmal nocturnal hemoglobinuria (PNH) [50]. In this acquired hemolytic disorder, the absence of complement regulators on clonal populations of blood cells renders affected erythrocytes highly susceptible to complement attack and MAC-mediated intravascular hemolysis [51, 52]. While treatment options had traditionally been limited, the approval of eculizumab in 2007 changed the management of PNH dramatically as the blockage of C5 activation and MAC formation largely impaired hemolysis. PNH thereby quickly rose to the status of the defining example of complement-mediated diseases. Yet although PNH is indeed among the most complement-driven disorders, it is hardly representative of complement’s broad involvement in clinical complications. In PNH, extrinsic stimuli such as bystander activation during infection act as initiator and the resulting pathomechanism is largely restricted to a single pathway (i.e., the AP) and effector (i.e., MAC). In contrast, most of the other complement-mediated disorders involve complex damage sensing events with parallel initiation of several pathways, which results in the generation of multiple effectors and extensive crosstalk.

**Fig. 2:**
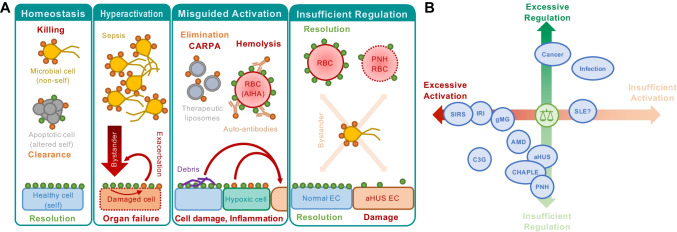
Principal mechanisms of complement involvement in disease. **A** The reaction of complement toward different surfaces is largely driven by the sum of stimulatory (orange circles) and regulatory entities (green circles). Under physiological conditions, this enables immune surveillance by forcefully removing microbial intruders and silently clearing endogenous threats while sparing healthy host cells. However, hyperactivation of the system by massive influx of bacteria (sepsis) may lead to strong bystander attack of host cells that overpower the regulatory capacity, while the inflicted cell damage may exacerbate the response. Biomaterials (e.g., liposomes), auto-antibodies (e.g., in AIHA), cell debris (e.g., atherosclerotic plaque), or hypoxia-induced damage patterns may trigger a misguided complement response that induces cell damage (e.g., reperfusion injury), inflammation, and/or adverse crosstalk reactions. Finally, insufficient regulation on host cells may increase their vulnerability to complement attack (e.g., by bystander activation). **B** Most complement-related conditions are driven by excessive activation and/or insufficient regulation of the complement response. However, exploitation of complement regulation is also observed as part of the immune invasion strategy of many pathogens and cancer cells

It is important to realize that the broad sensory capacity of complement of non-, damaged-, and altered-self surfaces, and its upstream positioning in defense reactions, renders complement a likely contributor to clinical conditions that involve exposure to foreign cells and materials (e.g., transplants or hemodialysis membranes) or altered endogenous surfaces (e.g., atherosclerotic plaque, malignant cells, damaged tissue) [3]. At the same time, it is quite rare that complement constitutes the sole or even dominant driver of disease, as in the case of PNH, but mostly acts as initiator, contributor, and/or exacerbator. Disorders with complement involvement therefore range from local to systemic and acute to chronic, can affect different organs and may exert highly distinct mechanisms. They are often affected by the genetic constellation of a patient’s complement components (sometimes referred to as ‘complotype’), age and environmental factors. Similar to the physiological processes discussed above, it is the sum of all forces that define the role of complement in a disorder. And while it is impossible to dissect complement’s involvement for each disease or even patient, complementopathy typically boils down to a few conceptual mechanisms: excessive activation, misdirected activation, insufficient regulation or, in rare cases, unwarranted regulation (Fig. 2).

Excessive activation is usually the base of (hyper-)acute inflammatory disorders and not necessarily linked to a specific complotype but often involves intense and highly damaging crosstalk with other defense systems. Appropriate activation triggers such as pathogen- or damage-associated molecular patterns (PAMPs or DAMPs, respectively) serve as inducers, but at such an extent that the fulminant complement activation results in host tissue damage and propagation of a vicious thromboinflammatory cycle. The timeliest example of such a disorder is COVID-19, the clinical manifestation of an infection with the Sars-CoV-2 virus that started a pandemic in 2020. Patients suffering from severe forms of COVID-19 show activation of several defense pathways that result in life-threatening inflammatory and thrombotic complications. Complement activation has been identified as a contributing factor [53, 54], although it is not yet clear whether complement is a cause of or reaction to the situation and whether the complement activation would be induced by the virus, ensuing cell damage or crosstalk mechanisms. The role of complement in other systemic inflammatory response disorders (SIRS) has been investigated more extensively [55–57]. Sepsis is possibly the condition most closely related to severe COVID-19 due to the initiating role of microbial particles. The sudden presence of PAMPs produces an excessive complement response with bystander damage of host cells, which may be exacerbated by complement activation via newly exposed DAMPs (i.e., an acquired positive feedback loop; Figs. 1, 2). Independently of the initial infectious trigger, the cell-destructive activities of complement and other pathways may eventually lead to tissue damage, multi-organ failure, and death. While several pathways and effectors may be involved in the process, the release of the anaphylatoxin C5a and its signaling via C5a receptor 1 (C5aR1) have been most closely linked to an adverse outcome [58]. In trauma, the exposure to microbes but also the inflicted tissue damage and/or hypoxia after injury may cause SIRS and negatively affect the clinical prognosis.

Similar to the case of SIRS, disorders of misdirected complement activation may occur despite a perfectly functioning complement system. Complement shows an ‘appropriate’ response to an inappropriate target. The exposure of foreign bodies such as transplants, implants, and other biomaterials are prominent examples. In transplantation medicine, incompatibility reactions by anti-HLA or -ABO antibodies that trigger the CP result in acute rejection, whereas the exposure of DAMPs after hypoxia may cause ischemia–reperfusion injury and damage of the donor organ [59]. Most attention in this area has been directed to solid organ transplantation, yet the same mechanisms may apply to cell transplantation or cell therapies, including CAR-T cell treatments in oncology, where inflammatory reactions up to cytokine storms are observed as adverse reactions [60]. Complement-mediated responses to biomaterials may be initiated by surface accumulation of complement-inducing molecules such as immunoglobulins or direct adsorption and conformational activation of complement components such as ficolins or C3. Whereas a misdirected complement activation is most obvious in the case of foreign body exposure, several autoimmune and age-related disorders may be counted to this category as well since they are also based on an unwanted reaction of a well-functioning complement system. As the formation of autoantibody clusters is a typical denominator of autoimmune diseases, CP-mediated complement activation is expected to be common in this disease class. So far, however, only few autoimmune conditions are considered complement-driven disorders. Autoimmune hemolytic anemia (AIHA), cold agglutinin disease (CAD) (summarized in this excellent review [61]), anti-acetylcholine receptor antibody-positive generalized myasthenia gravis (gMG) [62], anti-neutrophil cytoplasmic autoantibody (ANCA)-associated vasculitis [63], and anti-aquaporin-4 antibody-positive neuromyelitis optica spectrum disorder (NMOSD) [64] are examples that reached both clinical and therapeutic interest. In age-related diseases, it is the often-slow accumulation of cellular debris or misfolded protein plaque that induce complement activation. It is assumed that complement may initially hold disease progression in check by exerting its housekeeping function and contributing to the elimination of the waste products. Once the inducing structures cannot be removed any longer, however, the role of complement turns to the worse by causing tissue damage and inflammation. Age-related macular degeneration (AMD) has a particularly strong association to complement activation, although the disorder appears to be driven by insufficient regulation rather than misdirected activation. Conversely, direct complement activation was demonstrated on amyloid fibers and atherosclerotic plaque [65] but the impact of complement on the progression of Alzheimer’s disease or atherosclerosis is still debated.

In a majority of conditions, complement involvement may be driven, or at least largely influenced, by an imbalance between complement activation and regulation. Genetic variations, including primary deficiencies, deletions, polymorphisms, and mutations, are often the underlying cause [66]. For example, polymorphisms that reduce the activity of membrane-bound and soluble complement regulators are associated with AMD (see above) but also renal diseases such as atypical hemolytic uremic syndrome (aHUS) or C3 glomerulopathy (C3G). Interestingly, both conditions are also influenced by autoantibodies as disease-modulating factors yet with distinct mechanisms and consequences. In the case of aHUS, endothelial cell damage by complement-independent triggers is exacerbated by complement due to insufficient regulation on the cell surface. Autoantibodies against the C-terminus of FH, which is mediating self-cell recognition, may drive the disease by preventing the regulatory function of FH on the cell surface. In contrast, autoantibodies associated with the progression of C3G typically bind to and stabilize convertases or block the regulatory N-terminus of FH. Alongside increased activation on cell surfaces, these antibodies also impair the control of solution activation, thereby leading to a depletion of C3 in circulation and formation of dense C3b deposits in the renal tissue. FHRs and/or pentraxins are increasingly associated with complement disorders that are mediated by imbalanced complement activity. The previously mentioned PNH may be considered an extreme case of dysregulation. Most nucleated cells express a panel of four membrane-bound complement regulators that act at the convertase/opsonin (i.e., CR1, CD46, CD55) or MAC level (i.e., CD59). Whereas erythrocytes naturally lack CD46, the impaired biosynthesis of GPI anchors in PNH patients also depletes affected erythrocytes from CD55 and CD59, leaving them vulnerable to largely uncontrolled complement attack [51, 67]. More recently, a clinical link between CD55 and enteropathy has been described, reporting a life-threatening gastrointestinal disorder manifested in the eponymous CD55 deficiency with hyperactivation of complement, angiopathic thrombosis, and protein-losing enteropathy (CHAPLE) syndrome [68].

Whereas insufficient complement regulation is the culprit in many disorders, increased complement-regulatory capacities may unfavorably impact tumor development as many cancer cells were found to express high densities of membrane-bound complement regulators as part of their evasion strategy [69]. However, the involvement of complement in cancer progression is far more diverse and complex, and appears to be dependent on the cancer type/model and other factors. In fact, cancer is perhaps one of the clearest conditions in which the complement system displays its notorious role as a double-edged sword (for a more detailed discussion see [70]). On the one hand, complement is often found at the forefront of the battle of the host against aberrant, tumorigenic cells, hence the pressure to increase complement regulation for tumor development. In addition, complement plays an important positive role in the success of various therapies, such as radio-therapy [71], and new therapeutic options with improved complement activating capabilities are being explored [72]. On the other hand, complement activation has been implicated to facilitate a favorable niche for tumor growth. For instance, complement activation may hamper the local anti-tumor immune response [73], with C5a in particular playing detrimental roles in tumor development by inducing a pro-tumorigenic microenvironment [74], contributing to metastatic sites [75], and improving tumor mobility [76]. Thus, a counterintuitive inhibition of complement might actually be beneficial against certain tumors [77]. This complex, dual role of complement in tumor development makes therapeutic interference extremely challenging and the specific tumor type and progression stage will likely dictate a tailor-made pro- or anti-complement approach.

Increased regulation is also part of the evasion repertoire employed by many pathogens, including bacteria, viruses, fungi, and parasites. They either expose surface structures that recruit soluble regulators such as FH to their surfaces, cover themselves with regulator-containing host membranes, or secrete regulator mimics. We refer to specialized reviews for an overview of the extensive arsenal pathogens use to avoid complement-mediated destruction [78, 79]. However, these strategies, tested and perfected by evolution, may serve as blue-prints or inspiration for the development of new complement therapeutics, and several new inhibitors and complement-targeting technologies based on the principles identified in pathogens are currently being explored [80, 81].

Hemolytic, renal, and ocular diseases typically take center stage when listing complement disorders with strong complement association. The apparent susceptibility of those cells and organs to complement attack can potentially be attributed to several factors, including a strong exposure to circulating complement, low levels of complement regulators on certain cell types, or the presence of an unprotected basement membrane. However, complementopathies can principally affect any organ and there may be numerous disorders that have not entered the radar due to their low prevalence, complexity, or occurrence in tissues that are less accessible to diagnostics or relevant disease models. Latter is particularly true for neurological disorders, which are commonly regarded a new and promising frontier for complement research and therapy. For instance, genetic and/or mechanistic associations of adverse complement activation in the central nervous system have shown for Alzheimer’s and Parkinson’s disease, where protein plaques have been shown to activate complement and fuel neuroinflammation [65, 82]. In schizophrenia, the influence of complement is becoming more clear, with an apparent central, but nut fully understood, role for the locally expressed complement inhibitor CUB and Sushi Multiple Domains 1 (CSMD1) (reviewed in [83]). In all those conditions, it remains unclear how much complement contributes to beneficial (e.g., clearance of debris and synapses) and adverse reactions, and whether the system can and should be therapeutically modulated. The therapeutic interest has largely been shifted toward neuromuscular disorders, in particular myasthenia gravis (MG), amyotrophic lateral sclerosis (ALS), and Guillain–Barré syndrome (GBS). Although only some forms of generalized MG are currently treated with anti-C5 antibodies [62], there are active development programs for ALS [84, 85] and GBS [86]. Another area of novel or renewed interest are metabolic diseases, most prominently diabetes mellitus. Whereas a role for complement in the autoimmune-driven development of diabetes may not come as a surprise [87], less obvious interactions between complement proteins and diabetic disease reveal a more intricate, complex relationship. For instance, sustained high glucose levels in diabetic plasma may impair the complement-regulatory capacity of surface expressed CD59 throughout the body, leading to related complications [88], while intracellular CD59 isoforms are reported to be required for insulin secretion [89]. Furthermore, the anaphylatoxin C3a, generated through AP activation, seems to support pancreatic β cell homeostasis [90], which is of particular interest in obesity-related diabetes as adipocytes are the main producers of FD. While these are only a few examples, it indicates complement and metabolic diseases are affecting each other in expected and unexpected ways.

## One size does not fit all: extended options in therapeutic complement modulation

Drug discovery and development is a tedious and time-consuming process even under ideal circumstances, which often takes one or two decades from target validation to an approved drug. In the case of complement-targeted therapeutics, the time from associating complement with disease and the introduction of the first complement-specific drug spanned almost a century. The reasons for this slow progress have been manifold and include the initially limited understanding about complement mechanisms in health and disease, safety concerns about inhibiting a host defense pathway, and the challenging identification of indications with both well-defined complement involvement and suitable market size. Initiatives such as the orphan disease act partially relieved the dilemma as it allowed Alexion to develop their anti-C5 program for PNH, an ultra-rare disorder with a strongly complement-driven pathomechanism. The approval of eculizumab in 2007 presented a watershed moment for PNH patients who had access to an efficient therapy, for the company due to the commercial success of the program, and for researchers and clinicians who could finally benchmark the hypothetical considerations about function and safety in a clinical setting. Indeed, the past 15 years of anti-C5 therapy showed that the approach is generally safe and well-tolerated but that the risk of severe meningococcal infections needs to be tightly controlled by vaccination and reserve antibiotic strategies [5]. Importantly, the safe use in PNH enables a gradual extension of eculizumab’s indication spectrum, which meanwhile includes aHUS, gMG, and NMOSD. In addition, off-label use and clinical trials of the drug provide important insight into the benefit and limitations of therapeutic C5 inhibition, for instance most recently in the CHAPLE syndrome [91]. The eculizumab story, while successful and seminal for the field, also has some problematic aspects, though. Firstly, the focus on rare diseases and protection by the orphan disease act enabled a premium pricing model, with annual treatment costs in the $500,000 range, that imposed a significant burden on the healthcare system and limited accessibility to the drug in many countries; in this context, there is hope that the growing introduction of eculizumab biosimilars (and alternative therapeutics mentioned below) may provide enough market pressure to reduce cost. Moreover, Alexion’s tight control of the market and patient groups also provided a challenge for companies seeking to enter the PNH field. Indeed, the second complement-specific drug was only introduced in 2018, some 10 years after eculizumab, and was produced by the same company (Alexion) and bound to the same target (C5) and even epitope. What ravulizumab (Ultomiris) primarily improved upon was the dosing frequency, since changes in the antibody structure improved the plasma half-life and allowed for 8-week instead of 2-week dosing intervals [92, 93]. What had not been resolved, however, was the target diversity. When considering the broad and highly diverse disease involvement of complement as delineated above, it becomes evident that a single inhibitory strategy will not be applicable to all diseases. Indeed, some clinical trials with eculizumab (e.g., in AMD or C3G) did not reach the clinical endpoints. A greater diversity regarding targets, treatment modalities, and application routes is therefore critically needed.

**Fig. 3:**
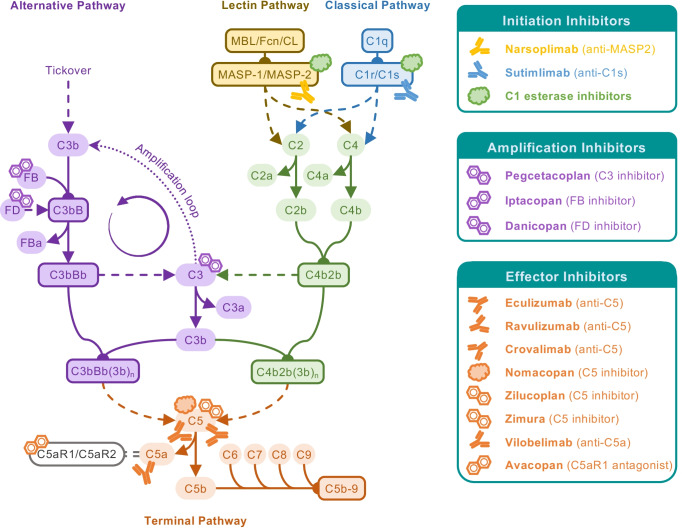
Complement-targeted therapeutics in the clinic or in late-stage development. Following eculizumab, the first complement-targeting therapeutic approved for a complement-driven disease (PNH), similar and novel therapeutic strategies are now at the final stage of development and on the verge of entering the clinic. Various inhibitors and biologicals act on C5, expanding on the success of eculizumab while fine-tuning the therapeutic strategy by specifically targeting C5a or its receptor (e.g., interfering with anaphylatoxin responses, while leaving MAC formation intact). Of special interest will be pegcetacoplan, as the first approved complement therapeutic that is acting more upstream within the cascade, closely followed by activation pathway-specific inhibitors. Of special note are preparations of C1 esterase inhibitor, a serine protease inhibitor which has been long used in the treatment of hereditary angioedema, unrelated to its effect on the lectin and classical activation pathways. With the rise of complement-targeting therapeutics, C1 esterase inhibitor have gained renewed interest as potential complement therapeutic that is easily accessible for the clinic. Furthermore, additional biologicals acting on the activation pathways are being explored, with narsoplimab (anti-MASP-2) and sutimlimab (anti-C1s) being the most advanced in development

Fortunately, the clinical and commercial success of eculizumab instigated a new confidence in complement inhibition as a therapeutic approach and many big and small pharmaceutical companies initiated corresponding development programs. While initially focusing on tried territory, with a strong emphasis on C5-targeted and PNH-directed approaches, the spectrum of clinical candidates has meanwhile reached an impressive diversity regarding targets (i.e., proteins of the CP, LP, amplification loop, and effector pathways) and modalities (i.e., small molecules, peptides, proteins, antibodies, siRNA, aptamers, etc.) (Fig. 3). The first fruits of these efforts have finally become palpable with the FDA approval of pegcetacoplan (Empaveli, Apellis), which targets C3 and thereby introduces the second class of complement-specific inhibitors (for a brief historical overview see [94]). Pegcetacoplan is a PEGylated peptide of the compstatin family of C3 inhibitors, originating from academic drug discovery at the University of Pennsylvania, which inhibits the activation of C3 by convertases and thereby affects opsonization and most effector generation. In the context of PNH, for which pegcetacoplan has been approved, inhibition at the C3 level may confer benefits over C5 inhibition since the impaired opsonization should also prevent potential extravascular hemolysis by immune cell recognition and breakthrough hemolysis caused by ongoing formation of C5 convertases. Indeed, in phase 3 studies pegcetacoplan showed superiority over eculizumab treatment in PNH [95]. Similar to the case of anti-C5 therapy some 15 years ago, the approval of pegcetacoplan presents an important clinical validation of the efficacy and safety of C3-directed therapies, especially since concerns had been raised about such broad and upstream intervention. In clinical studies, even long-term treatment with pegcetacoplan was well-tolerated. An increased risk for certain infections is again a point that requires careful monitoring; in addition to *Neisseria meningitidis*, patients receiving pegcetacoplan also need to be vaccinated against *Streptococcus pneumoniae* and *Hemophilus influenzae* type B. It will be interesting to observe how pegcetacoplan performs in clinical practice and whether a similar broadening of indication areas as in the case of eculizumab will take place. Indeed, clinical trials with pegcetacoplan are ongoing in AMD, C3G, CAD, amyloid lateral sclerosis, and hematopoietic stem cell transplantation-associated thrombotic microangiopathy (HSCT-TMA). Other compstatin-based programs are in development by Apellis and Amyndas Pharmaceuticals, latter of which uses AMY-101, a next-generation compstatin analog with largely improved activity, target residence, and pharmacokinetic properties, as clinical candidates [96]; AMY-101 is currently evaluated in clinical trials for periodontal disease and COVID-19 with plans for C3G, PNH, and ABO-incompatible kidney transplantation [94].

Yet there are even more candidates in late-stage trials or approval registration that cover various stages of complement activation. With sutimlimab and narsoplimab, two antibodies that block the initiation proteases of the CP and LP, respectively, have completed phase 3 trials. The anti-C1s antibody sutimlimab is developed by Sanofi as a treatment option for CAD [97] and, after the initial registration filing was returned by the FDA, Sanofi is currently preparing for refiling after acquiring another set of supportive phase 3 data. Omeros’ narsoplimab is an antibody blocking MASP-2 that is evaluated in phase 3 trials for aHUS, HSCT-TMA, and IgA nephropathy [98–100]. Based on positive results in HSCT-TMA, a biologics license application is currently reviewed by the FDA. In this context, it has to be noted that therapeutics with activity against CP and LP proteases have been available to the clinic even before the introduction of eculizumab. However, those plasma-purified or recombinant preparations of the physiological regulator C1 esterase inhibitor (C1-INH) are not complement-specific and also block proteases of the coagulation and kinin system, and are thus-far only approved for a disease with no major complement involvement (i.e., hereditary angioedema) [101]. The prospect of having initiation pathway-specific inhibitors as part of the therapeutic arsenal is therefore exciting. Whereas compstatin-based C3 inhibitors have a major impact on the amplification loop yet impair C3 activation more broadly, AP-specific inhibitors have meanwhile also researched late-stage development. These typically target one of the two serine proteases involved in C3 convertase formation. Danicopan (Alexion) is a small molecule inhibitor of factor D (FD), a protease critical for the functional assembly of the C3bBb complex and considered the rate-limiting step of the AP [102]. Currently, Alexion is developing danicopan as add-on therapy to eculizumab/ravulizumab in PNH, for patients remaining transfusion-dependent due to extravascular hemolysis under anti-C5 treatment. While this indication focus may prove limiting when considering the availability of pegcetacoplan, the oral bioavailability of danicopan may open opportunities in other indications. Oral application is also the administration route of choice for the factor B (FB) inhibitor iptacopan (Novartis) [103]. As the Bb fragment of FB constitutes the enzymatic principle of the C3 convertase, iptacopan also inhibits already assembled convertases, which may provide a particular benefit over FD inhibitors in C3G, which is driven by highly stable convertases.

Despite the clinical availability of anti-C5 antibodies, C5-induced effector generation and function remains an important and highly active target area. Anti-C5 antibodies with distinct epitopes, pharmacokinetic profiles, and administration routes are developed by several companies. Among those, crovalimab (Roche) has progressed the farthest with ongoing phase 3 trials for PNH. Due to its favorable subcutaneous bioavailability, crovalimab may be self-administered by the patient [104]. In addition to antibodies, other C5-targeted modalities begin to emerge. Nomacopan (Akari), a tick-derived protein inhibitor with dual activity for C5 and leukotriene B4 [105], is in phase 3 trials for pediatric HSCT-TMA with another phase 3 trial for the autoimmune disease bullous pemphigoid in preparation. The macrocyclic peptide zilucoplan (UCB) [106], on the other hand, has entered phase 3 trials for gMG; similar to crovalimab in PNH, zilucoplan may confer a benefit over current anti-C5 therapy in gMG by allowing for patient self-administration [107]. Of note, zilucoplan did not show meaningful efficacy in phase 2 trials for immune-mediated necrotizing myopathy [108], which indicates that not all autoimmune disorders benefit from C5- or even complement-targeted therapy. Finally, the C5-binding aptamer zimura (Iveric Bio) has a therapeutic focus on ocular disease, with ongoing phase 3 trials for AMD and phase 2 studies in Stargardt disease [109]. Whereas C5 inhibition simultaneously prevents MAC formation and C5a release, the therapeutic impairment of a single effector arm may be effective and sufficient in some diseases. While MAC-directed approaches are in earlier development stages, inhibitors targeting the C5a-C5aR1 signaling axis have now reached late-stage development. Avacopan (Chemocentryx/Vifor), a low molecular weight C5aR1 antagonist that is administered orally, has completed phase 3 trials for ANCA-associated vasculitis [110] and has filed a new drug application with the FDA. The antibody vilobelimab (InflaRx) targets the effector rather than the receptor by binding and neutralizing C5a [111]. Phase 3 studies of vilobelimab are conducted or planned for severe COVID-19 and for the skin disease hidradenitis suppurativa, respectively, with ANCA vasculitis and other autoimmune disorders explored at earlier stages.

In this brief summary, we focused on late-stage development programs and refer to recent reviews for a broader overview of the rich and promising pipeline of therapeutic complement modulators [4, 112]. Yet even this short glimpse reveals that the field of complement-targeted drug discovery has seen a remarkable transformation and finally reached maturity. The increasing diversity regarding target and modalities is expected to benefit the patients, the healthcare system and the clinical and research community. It not only allows for a clinical validation and optimization of therapies in established complement-mediated diseases but also the extension into new indication areas and the combination of treatment modalities in complex, acute-phase diseases. While safety concerns need to be taken seriously and require monitoring and mitigating strategies, current and previous evaluations of complement inhibitors of all stages rarely indicated severe adverse effects. Rather, it was a lack of efficacy that typically led to attrition of some earlier candidates. Alongside technical and mechanistic issues, the complexity and diversity of some of the explored indication and the definition of suitable endpoints may have been the real culprit in many cases. The evaluation of the anti-FD antibody lampalizumab in AMD serves as a prominent example [113], in which disappointing efficacy assessments led to a halt of phase 3 trials and an abandonment of the program. Although a role of complement in AMD progression is established, complement is not the driver of disease in all patients. Even in complement-sensitive forms of AMD, similar manifestations may be caused by distinct molecular mechanisms and may require therapeutic approaches. Careful stratification of patient groups, and sensitive monitoring of complement activation profiles, will therefore be essential for matching indications, patient groups, and treatment strategies.

## Conclusion

The catalogue of clinical conditions with confirmed complement contribution has been growing continuously over the past decades. Considerable advances in the understanding of complement functions and crosstalk and in the diagnosis of disease, including the emergence of genome-wide association studies, have essentially contributed to this development. Paradoxically, the general progress in modern medicine may have also played its share to the growing prevalence of complement disorders as we increasingly expose our bodies to foreign materials (e.g., transplants, implants, cellular and liposomal therapeutics) and since complement is increasingly challenged with cell debris as we grow older. Yet it likely has been the successful introduction of complement-targeted therapies that has served as the strongest accelerator for the recognition of complement as disease contributor and therapeutic target. After an initial ‘hype’ with high hopes and disappointing outcomes in clinical studies, followed by a valuable consolidation phase to realize that the complexity of complement’s disease involvement cannot be addressed by a ‘one-size-fits-all’ approach, we finally see the fruits of the long-standing efforts in research and development coming to full blossom. The approval of pegcetacoplan has marked an important milestone and the diverse set of late-stage clinical candidates raises the very realistic hope that we may soon tailor the complement-targeted treatment option to the right indications and patients. It is expected that this extension of the therapeutic arsenal, alongside improved diagnostic capacities and a growing awareness of complement among the clinical community, will lead to appearance of even more disorders on the map of complementopathies and of improved therapeutic options for many patients. Complement may not be a ‘novelty’ but sure has opened new insights and avenues for biomedical research and disease management.
